# Effect of exercise training and dopamine agonists in patients with uremic restless legs syndrome: a six-month randomized, partially double-blind, placebo-controlled comparative study

**DOI:** 10.1186/1471-2369-14-194

**Published:** 2013-09-11

**Authors:** Christoforos D Giannaki, Giorgos K Sakkas, Christina Karatzaferi, Georgios M Hadjigeorgiou, Eleftherios Lavdas, Theodoros Kyriakides, Yiannis Koutedakis, Ioannis Stefanidis

**Affiliations:** 1 Department of Nephrology, School of Medicine, University of Thessaly, Larissa, Greece; 2 Department of Neurology, School of Medicine, University of Thessaly, Larissa, Greece; 3 Department of Radiology, School of Medicine, University of Thessaly, Larissa, Greece; 4 Department of PE and Sport Science, University of Thessaly, Trikala, Greece; 5 Institute for Research and Technology Thessaly, Center for Research and Technology Hellas, Trikala, Greece; 6 Department of Life & Health Sciences, University of Nicosia, Nicosia, Cyprus; 7 The Cyprus Institute of Neurology and Genetics, Nicosia, Cyprus

**Keywords:** Extramyocellular lipids, Lean body mass, Muscle atrophy, Quality of life, Sleep

## Abstract

**Background:**

Restless Legs Syndrome is very common in hemodialysis patients however there are no comparative studies assessing the effectiveness of a non-pharmacological treatment to a classical treatment on parameters related to syndromes’ severity and quality of life.

**Methods:**

In this randomized, partially double blind, placebo controlled trial, thirty two hemodialysis patients with restless legs syndrome were randomly assigned into three groups: 1) the exercise training group (N = 16), 2) the dopamine agonists group (ropinirole 0.25 mg/d) (N = 8) and 3) the placebo group (N = 8). The intervention programs lasted 6 months. Restless Legs Syndrome severity was assessed using the international severity scale, physical performance by a battery of tests, muscle size and composition by computed tomography, body composition by Dual Energy X Ray Absorptiometry, while depression score, sleep quality, daily sleepiness and quality of life were assessed through questionnaires.

**Results:**

Exercise training and dopamine agonists were effective in reducing syndrome’s symptoms by 46% (P = 0.009) and 54% (P = 0.001) respectively. Within group changes revealed that both approaches significantly improved quality of life (P < 0.05), however, only the dopamine agonists significantly improved sleep quality (P = 0.009). Within group changes showed a tendency for lean body mass improvements with dopamine agonists, this reached statistical significance only with the exercise training (P = 0.014), which also reduced fat infiltration in muscles (P = 0.044) and improved physical performance (P > 0.05) in various tests. Between group changes detect significant improvements with both exercise and dopamine agonists in depression score (P = 0.003), while only the dopamine agonist treatment was able to significantly improve sleep quality, compared to exercise and placebo (P = 0.016).

**Conclusions:**

A 6-month exercise training regime was as effective as a 6-month low dosage dopamine agonist treatment in reducing restless legs syndrome symptoms and improving depression score in uremic patients. Further research is needed in order to show whether a combination treatment could be more beneficial for the amelioration of RLS.

**Trial registration:**

NCT00942253

## Background

Restless Legs Syndrome (RLS) is a highly prevalent sensory-motor disorder in the hemodialysis (HD) population [1]. Dopamine agonists (DA) is the choice medication and is effective in the reduction of RLS symptoms in patients suffering from idiopathic RLS [2]. However, limited data exist regarding the therapeutic effectiveness of DA in HD patients with a secondary form of the syndrome [3,4], called uremic RLS.

Recently published data indicate that patients with uremic RLS are subjects of increased muscle atrophy compared to RLS free counterparts [5]. Muscle atrophy and the total lean body mass receives special concern from the nephrologists as it has been related to high mortality in the HD patients [6].

Even though, DA are the treatment of choice for the amelioration of RLS symptoms, side effects and augmentation phenomena have been commonly reported in the literature [7]. Evidence from non-RLS individuals indicated that dopaminergic agents may enhance physical performance by delaying the onset of muscle-related fatigue [8]. On the other hand, acute [9] or chronic [10] exercise training has been used successfully in HD patients as a means of ameliorating RLS symptoms concomitantly improving aspects of quality of life (QoL). It is known that both DA and exercise training can ameliorate RLS symptoms however it has not yet been elucidated whether treatment with DA could improve parameters related to physical performance and muscle size in patients with uremic RLS. The primary aim of the current study was to compare the changes across groups in the amelioration of RLS symptoms in HD patients with RLS. The secondary aim was to assess the changes across groups in aspects related to QoL and overall life prognosis such as muscle size, body composition and physical performance.

## Methods

### Ethics statement

The study was approved by the Human Research and Ethics Committee of the University of Thessaly, and by the bioethics committee of the University General Hospital of Larissa, Greece. All patients gave their written informed consent prior to study participation.

### Study population

All patients were recruited from the hemodialysis unit of the University Hospital of Larissa (UHL), Greece. The study was performed from September 2007 to October 2009. All study measurements were performed at the University Hospital of Larissa, Greece. RLS was diagnosed by a single RLS specialist-neurologist using the standard criteria of the International RLS Study Group (IRLSSG) [11]. Furthermore, the patients were included in the study if they had at least one RLS episode per week excluding the episodes that took place during the dialysis session. RLS severity was assessed using the IRLSSG severity rating scale [12].

The inclusion criteria for the study were: dialysis for at least three months or more with adequate dialysis delivery and with stable clinical condition. In addition, all patients should have RLS and none of them should have been treated with any medication for RLS prior to the study.

Exclusion criteria included diagnosed neuropathies (n = 3, clinically examined by a neurologist) or reasons for being in a catabolic state (n = 2, opportunistic infections and active inflammation), within 3 months prior to the start of the study, or with C-reactive protein blood levels above 3.0 mg/l (n = 1), unable to exercise (n = 3) or refuse to participate for personal reasons (n = 4). None of the recruited patients were engaged in any systematic exercise training programme. A specialized in RLS neurologist examined the patients in order to assess any potential augmentation phenomena during the study period, using standard criteria [11].

**Figure 1:**
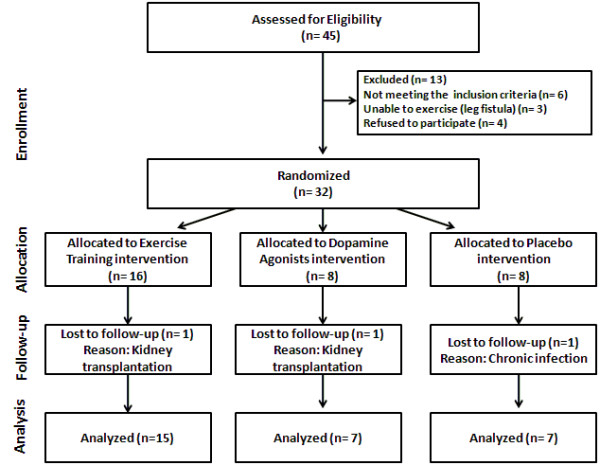
**Patient recruitment flow diagram.** Disposition of the patients into the three groups as follows: aerobic exercise training group, dopamine agonists group and placebo group.

Over all, thirteen patients fulfill the exclusion criteria (Figure 1) while thirty-two stable HD patients were enrolled to the study.

Patients were randomly assigned, in a 2-1-1 fashion, in three groups. The first group received 6 months of intradialytic exercise training (N = 16 – Exercise group), the second group received the classical RLS treatment with DA (ie Ropinirole) (N = 8 – Dopamine agonist group) while the third group received matching placebo pills (N = 8 – Placebo group). All three interventions lasted for six months in order to maximize the exercise effect and to minimize the well-known placebo effect seen in RLS patients. Both the patients and the investigators were blinded to the type of the medication used in the second and third group (double-blind design).

### Medication

The DA used was ropinirole (Adartrel®, GlaxoSmithKline, UK). It was powdered and inserted in an empty capsule (the rest was filled with plain flour), in a 0.25 mg/dose, which was maintained stable until the end of the study. We consciously decided not to perform a dose titration in order to minimize any augmentation phenomena that could affect the study outcomes. Placebo was made by plain flour filled in the same type of capsules. Patients were instructed to receive their capsules once daily, 2 hours before bedtime. Both the ropinirole and the placebo capsules were made at the UHL pharmacy and their packaging was identical. The efficacy of DA or placebo on RLS symptoms severity was evaluated by the IRLSSG scale [12].

### Exercise training

The exercise training took place 3-times per week during the HD session, for six months and consisted of cycling in a recumbent cycle ergometer (Model 881 Monark Rehab Trainer, Varberg, Sweden) at an intensity of 60-65% of the patient’s maximal exercise capacity (in Watts), which was estimated through during a previous HD session [13]. The exercise intensity was readjusted on a monthly base.

### Hemodialysis procedure

The patients underwent the HD therapy (Fresenius 4008B, Oberursel, Germany) 3 times/week with low flux, hollow-fiber dialysers and bicarbonate buffer. The HD session lasting approximately 4 hours. An enoxaparin dose of 40–60 mg was administered intravenously before the beginning of each HD session. EPO therapy was given after the completion of HD session in order to normalize hemoglobin levels within 11–12 (g/dL).

### Physical performance

The patient’s physical performance levels were assessed via a battery of specific tests, including the North Staffordshire Royal Infirmary (NSRI) walk test [14], two Sit-to-Stand tests (STS-5 STS-30 and STS-60) and two gait speed tests (normal and fast walk) [15].

### Body and muscle composition

The patient’s whole body and regional fat and lean body mass were measured by a dual energy x-ray absorptiometer (DEXA) system (Lunar model DPX Madison, WI), as previously described [16].

Muscle size and composition were assessed by analyzing images collected by a Computed Tomography (CT) system (Philips, Tomoscan SR5000) [17,18]. Muscle cross sectional area (CSA), subcutaneous adipose tissue (SAT) and extramyocellular lipids (EMCL) accumulation were assessed by images collected using the same CT system by collecting six images of 2 cm apart at the larger girth of the right thigh for each patient. Image analysis of the CT slices was performed using a customized software program written in IDL (Interactive Data Language Research Systems, Inc., Boulder, CO). This software, based on variations in signal intensity, allowed for the quantification of fat, muscle and miscellaneous components (connective tissue, fascia, intramuscular fat) expressing the data as area (cm^2^) and percentage of tissue composition (fat%, muscle% and miscellaneous%).

### Questionnaires

All questionnaires were completed with the interview method, by experienced personnel. The patient’s subjective quality of life outcomes were evaluated by using a SF-36 Health Survey version [19]. The scores in the SF-36 can range from 0 to 100, while higher scores indicate better health status. The 8 multi-item scales of the SF-36 were summarized into two components (mental and physical component respectively) [19].

The patients’ depression levels were assessed by using the self-rating depression scale developed by Zung [20]. The cut-off point for the diagnosis of clinical depression in the Zung scale is above 50.

The Epworth sleepiness scale was used to assess the daily sleepiness level of the patients [21]. Briefly, this scale consists of questions referring to eight situations. The patients are asked to rate on a scale of 0 up to 3 how likely they would be to doze off or fall asleep during the 8 situations. Outcome scores can range from 0 to 24. A score above 10 is considered to be pathological sleepiness.

Finally, a weekly sleep diary was used to evaluate the patient’s quality of sleep. The diary was adapted from the University of Massachusetts Medical School website (http://healthnet.umassmed.edu/mhealth/WeeklySleepQuestionnaire.pdf). Briefly, the sleep diary contained questions regarding how often during the previous week HD patients experienced any of the following: (1) difficulties falling asleep, (2) number of nocturnal awakenings, (3) difficulties remaining asleep, (4) the sensation of waking-up tired and fatigued, (5) day time stress and (6) how often did they feel refreshed after the night’s sleep. The sleep diary was scored as follows: ‘never’ (0 points), ‘1–2 times a week’ (1 point), ‘3–5 times a week’ (2 points), ‘6–7 times a week’ (3 points). For question number 6 the scoring was reversed with 3 points for the answer ‘never’ , and 0 points for the answer ‘6–7 times a week’. The sleep diary score was calculated as the sum of the total points with the minimum at zero points and the maximum score at 18.

### Biochemical assessment

The biochemical examination of blood samples was performed at the clinical lab of the UHL under standard hospital procedures. A single-pool Kt/V was calculated from using the Daugirdas II equation [22].

### Statistical analysis

The baseline characteristics of the three groups were compared using one-way ANOVA. The changes within the three groups from baseline to the end of the 6-month intervention period were evaluated using paired t-tests while chi-square was used for categorical variables. The absolute changes between the three groups from baseline to the end of the 6-months intervention were evaluated via one-way ANOVA. Finally, Spearman rank correlation test was used to assess the relation between the examined variables. All statistical analyses were performed using the SPSS version 15.0 (SPSS Inc. Chicago, Illinois). Data in the text are presented as mean ± SD and the level for statistical significance was set at P ≤ 0.05.

Sample size calculations were conducted based in IRLS score values after pharmacological [IRLS 26(5) vs 11(9)] and exercise treatment [IRLS 26(6) vs 15(9)] from previous published work [10,23]. Even though the dose of the pharmacological treatment used for the power calculation [23] was higher compared to our study, the magnitude of change was almost identical (42% vs 45%). The resulting minimum required sample size was an average of 6 for the pharmacological approach and an average of 9 for the exercise approach for 2-sided type 1 and type 2 errors of 5%.

## Results

The study’s flow chart is presented in Figure 1. Briefly a total of forty-five patients were screened while thirty-two patients enrolled in the study and were randomly assigned in one of the three groups. Three patients dropped out for reasons unrelated to the study during the follow-up period making those who completed the study twenty nine.

The patient’s characteristics are presented in Table 1. No significant differences were observed in the patient’s baseline characteristics and none of these values changed after the 6-month intervention in all three groups (post data not shown) (P > 0.05).

**Table 1:** Patient’s characteristics data divided in three groups according to the assigned intervention

**Variables**	**Exercise group**	**Dopamine agonists group**	**Placebo group**
N	15	7	7
Female/Male	4/11	3/4	2/5
Age (yr)	56.4±12.5	55.7±10.4	56.8 ±16.5
BMI (Kg/m^2^)	27.0±3.6	27.4±5.6	25.3±1.7
Kt/V	1.2±0.2	1.3±0.1	1.2 ±0.0
Years in Hemodialysis	3.9±1.3	4.0±1.7	3.6±1.5
Iron (μg/dl)	58.6± 17.4	64.5±14.2	61.4±12.6
Hct	36.8±5.7	40.0±3.6	37.1±2.0
Hb (g/dL)	12.1±2.0	13.5±1.1	12.0±0.6

All data are mean ± SD. Abbreviations: *BMI* Body mass index, *Kt/V* Dialysis efficiency, *Hct* Hematocrit, *Hb* Hemoglobin.

The baseline score in the IRLS severity scale, Zung depression scale, sleep diary, daily sleepiness status and overall QoL score did not differ between the three groups. Both exercise and DA intervention were equally effective in reducing RLS symptoms (P = 0.012) and depression score (P = 0.003) compared to the placebo arm while the DA group was more effective in improving sleep score (P = 0.016) compared to exercise and placebo groups (ANOVA – between groups effect) (Table 2).

**Table 2:** Quality of life, depression, daytime sleepiness, sleep quality and RLS symptom’s severity data divided in three groups divided in three groups according to the assigned intervention

**Variables**	**Exercise group**	**Dopamine agonists group**	**Placebo group**
**Sleep Diary**			
Baseline	7.69±5.51	10.28±2.87	8.42±5.09
6-m post	7.30±4.13	5.85±3.71†	8.85±4.09
Δ Change	−0.71±3.22	- 4.42±3.10#¶	0.42±2.7
95% CI of Δ change	−2.49 to 1.07	−7.28 to −1.56	2.91 to −2.07
**Epworth Sleepiness Scale**			
Baseline	5.15±2.54	7.85±5.24	8.71±5.37
6-m post	5.15±2.47	5.42±1.98	8.42±4.92
Δ Change	−0.35±2.73	−2.42±5.38	- 0.28±4.19
95% CI of Δ change	−1.85 to 1.15	−7.39 to 2.55	−4.15 to 3.59
**Zung Depression Scale**			
Baseline	43.45±8.06	46.85±13.49	37.85±12.15
6-m post	35.84±6.38†#	39.42±4.35	43.71±11.17†¶
Δ Change	−8.28±7.91#	−7.42±10.34#	5.85±6.41
95% CI of Δ change	−12.60 to −3.91	−16.96 to 2.12	11.70 to −0.07
**IRLS score**			
Baseline	25.14±9.09	24.14±5.55	19.71±7.49
6-m post	13.42±11.28†	11.57±7.84†	18.57±10.65
Δ Change	−13.12±8.59#	- 12.57±5.31#	- 1.14±8.43
95% CI of Δ change	−17.87 to −8.37	−17.47 to −7.67	−8.93 to 6.65
**SF-36 MCS score**			
Baseline	61.1±22.0	39.1±23.8	68.1±19.1
6-m post	70.4±18.7	63.0±17.0†	65.0±21.9
Δ Change	9.3±26.7	23.8±14.1	3.1±9.8
95% CI of Δ change	24.1 to 5.4	36.9 to 10.7	5.9 to −12.2
**SF-36 PCS score**			
Baseline	64.9±18.6	48.7±21.0	64.4±22.5
6-m post	76.4±15.6†	68.8±19.2†	70.5±26.5
Δ Change	11.5±18.4	20.1±6.0	6.1±5.7
95% CI of Δ change	21.7 to 1.2	25.7 to 14.5	20.2 to −7.9

All data are mean ± SD. Abbreviations: *IRLS* International restless legs syndrome severity scale, *SF-36* Self reported quality of life questionnaire, *MCS* Mental component summary, *PCS* Physical component summary.

#Significantly different than placebo group.

¶Significantly different than exercise group.

**†**Significantly different from the respective baseline value.

DA and exercise training positively affected the SF-36-PCS score (P = 0.000 and P = 0.003), DA positively affected SF-36-MCS (P = 0.004), however, even though the improvements rich the 18%, 41% and 9% for the PCS and 15%, 61% and 4.5% (worsening) in the MCS in the Exercise, DA and Placebo groups, the Δ-changes values did not reach statistical significant levels (P < 0.05).

In addition, depression score appeared to be significantly improved (P = 0.002) in the exercise group after the 6 months, whereas in contrast, in the placebo group, a significant worsening of the score was detected (P < 0.05).

IRLS score was significantly reduced after the intervention with exercise or DA by 46% (P = 0.009) and 54% (P = 0.001) respectively, whereas no significant changes were found in the placebo group after the 6 months period (P = 0.732) (paired t-test).

The physical performance data are presented in Table 3. For the baseline, no significant differences were found between the three groups in all physical performance tests performed (P > 0.05). Exercise training significantly increased the patient’s performance in the NSRI (P = 0.023), STS-5 (P = 0.014) and STS-30 (P = 0.040) tests (P < 0.05), whereas no significant changes were observed in the DA and in the placebo groups (P > 0.05).

**Table 3:** Physical performance data divided in three groups according to the assigned intervention

**Variables**	**Exercise group**	**Dopamine agonists group**	**Placebo group**
**STS- 5 (sec)**			
Baseline	10.12±2.56	8.89±0.74	9.69±0.46
6-m post	8.24±2.34†	8.48±1.72	8.81±0.66
Δ Change	−1.75±2.32	- 0.41±1.63	- 0.88±0.76
95% CI of Δ change	−3.03 to −0.47	−1.91 to 1.09	−1.58 to −0.18
**STS-30 (rep)**			
Baseline	14.73±4.00	14.83±2.13	14.75±0.9
6-m post	17.84± 4.68†	14.50±2.58	17.25±3.2
Δ Change	2.96±4.87	- 0.33±2.33	2.50±2.51
95% CI of Δ change	5.65 to 0.27	−0.63 to −0.03	4.81 to −0.19
**STS-60 (rep)**			
Baseline	27.95±8.38	26.60±3.64	30.0±3.16
6-m post	32.50±9.34	27.40±7.40	32.0±5.35
Δ Change	4.26±8.23	0.80±4.65	2.00±3.26
95% CI of Δ change	8.77 to −0.25	5.09 to −3.49	5.0 to −1.0
**Normal Walk (sec)**			
Baseline	6.09±1.24	5.69±1.53	5.30±1.06
6-m post	5.81±1.49	6.14±1.63	5.38±1.09
Δ Change	−0.42±1.36	0.44±1.46	0.07±0.27
95% CI of Δ change	−1.17 to 0.33	1.78 to −0.90	0.31 to −0.17
**Fast Walk (sec)**			
Baseline	3.86±0.66	3.57±0.40	3.77±0.47
6-m post	3.65±0.75	3.56±0.40	3.57±0.41
Δ Change	−0.28±0.65	- 0.01±0.19	- 0.19±0.40
95% CI of Δ change	−0.63 to 0.07	−0.18 to 0.16	−0.55 to 0.17
**NSRI test (sec)**			
Baseline	84.46±21.35	70.10±20.46	52.48±14.24
6-m post	75.02±26.60†	67.68±20.78	53.44±17.13
Δ Change	−9.25±13.41	−2.41±6.52	0.96±4.01
95% CI of Δ change	−16.6 to −1.85	−8.41 to 3.59	4.65 to −2.73

All data are mean ± SD. Abbreviations: *STS-5* Sit-to- stand test 5-repetitions, *STS-60* Sit-to- stand test 60 seconds, *STS-30* Repetitions’ measurement at 30 seconds as part of the STS-60 test, *NSRI* North staffordshire royal infirmary test.

**†**Significantly different from the respective baseline value.

DEXA and CT data are presented in Table 4 and Table 5 respectively. Total lean body mass (LBM) appeared to be significantly increased after the exercise training (P = 0.014), however the Δ change value was not significantly different between the groups. The EMCL were significantly reduced after the exercise intervention (P = 0.044).

**Table 4:** Body composition data divided in three groups according to the assigned intervention

**Variables**	**Exercise group**	**Dopamine agonists group**	**Placebo group**
**DEXA-derived data**			
**Total Body Fat (%)**			
Baseline	30.9±9.0	31.9±11.2	26.0±5.4
6-m post	30.5±8.1	27.1±7.5	28.5±5.8
Δ Change	- 0.38±1.83	- 4.71±10.86	2.45±2.24
95% CI of Δ change	−1.39 to 0.63	−14.70 to 5.32	4.50 to 0.38
**% Legs Fat**			
Baseline	32.7±10.2	29.9±13.2	26.2±4.9
6-m post	32.3±9.3	25.1±8.9	28.5±5.2
Δ Change	- 0.37±2.71	- 4.86±11.03	2.27±6.50
95% CI of Δ change	−1.86 to 1.12	−15.05 to 5.28	8.27 to −3.73
**Total LBM (Kg)**			
Baseline	45.3±7.8	47.7±5.4	46.1±5.0
6-m post	46.7±8.3†	49.8±3.4	45.4±5.1
Δ Change	1.38±1.23	2.12±4.85	- 0.66±0.71
95% CI of Δ change	2.06 to 0.70	6.61 to −2.37	−1.31 to −0.01

All data are mean ± SD. Abbreviations: *LBM* Lean body mass.

**†**Significantly different from the respective baseline value.

**Table 5:** Muscle composition and size data divided in three groups according to the assigned intervention

**Variables**	**Exercise group**	**Dopamine agonists group**	**Placebo group**
**EMCL CSA (cm**^**2**^**)**			
Baseline	13.9±5.1	7.6±2.1	10.0±5.2
6-m post	11.2±5.8	6.8±3.6	9.4±2.0
Δ Change	−2.62±5.15	−0.79±3.33	−0.61±3.92
95% CI of Δ Change	−5.46 to 0.22	−3.86 to 2.21	−4.23 to 3.01
**EMCL (%)**			
Baseline	10.9±3.8	8.7±3.6	8.7±3.9
6-m post	8.2±3.5†	7.1±2.7	8.4±1.5
Δ Change	−2.70±3.90	−1.65±3.45	−0.21±3.54
95% CI of Δ Change	−4.85 to −0.55	−4.83 to 1.53	−3.48 to 3.06
**Muscle CSA (cm**^**2**^**)**			
Baseline	99.5±14.5	82.5±22.5	98.9±7.3
6-m post	106.8±24.4	89.4±24.5	95.8±2.0
Δ Change	7.32±16.03	6.93±17.66	- 3.15±7.87
95% CI of Δ Change	16.18 to −1.54	23.22 to −9.39	−10.42 to 4.12
**Muscle (%)**			
Baseline	87.9±3.7	85.7±7.2	88.1±3.9
6-m post	88.9±4.5	88.1±5.9	88.2 ±2.2
Δ Change	0.96±5.50	2.39±4.89	0.07±1.64
95% CI of Δ Change	4.00 to −2.04	8.90 to −2.12	1.58 to −1.44
**SAT CSA (cm**^**2**^**)**			
Baseline	142.8±57.8	88.7±46.7	103.6±28.4
6-m post	143.8±64.8	95.5±39.3	99.7±14.9
Δ Change	0.98±18.88	6.80±19.34	−3.95±17.97
95% CI of Δ Change	11.42 to −9.46	24.67 to −11.07	−20.55 to 12.65

All data are mean ± SD. Abbreviations: *EMCL* Extramyocellular lipids (fat infiltration), *CSA* Cross sectional area, *SAT* Subcutaneous adipose tissue.

**†**Significantly different from the respective baseline value.

The post exercise IRLS severity score was negatively correlated with the post exercise QoL score (r = − 0.719, P = 0.045) and the amount of fat infiltration in the muscles (EMCL, r = − 0.829, P = 0.021) post training. Finally, none of the patients reported any drug adverse reactions or augmentation phenomena from the three interventions.

## Discussion

Both low dose ropinirole and aerobic exercise training treatment were found to equally ameliorate RLS symptoms and improve depression score, while no side effects were reported in either treatment. However, only dopamine agonists improve subjective sleep quality while the placebo group reported a significant deterioration in the depression symptoms score after the obvious failure of the treatment.

The DA ropinirole is considered an effective approach for the amelioration of RLS symptoms in both idiopathic [2] and uremic [4] RLS patients, and is the treatment of choice despite some reports of augmentation problems when dosage increases [7]. Still, there is recent evidence that aerobic exercise training could effectively reduce RLS symptoms, in both categories of RLS patients [9,24].

Our study, comparing these two approaches, showed that both aerobic exercise training and the starting dose of ropinirole treatment could equally ameliorate the severity of RLS symptoms (by 46% and 54% respectively) compared to placebo.

To the best of our knowledge, this is the first study to compare the classical RLS pharmacological treatment over an alternative but very promising approach such as exercise training. The ropinirole dosage used in our trial was the minimum approved in order to effectively reduce the RLS symptoms and at the same time avoid augmentation phenomena [2]. The low dosage employed could explain the lack of substantial difference in the reduction of IRLS score between the classical treatment and the exercise training. It should be noted that improvements in other studies were reported to reach 75% in the IRLS severity scale, using however dosages that ranged from 0.75 mg/day (already higher dosage than ours) up to 2 mg per day [4]. The uniqueness of the current study is that by using the lower ropinirole dosage we were able to avoid any augmentation symptoms and confer clinical benefit that was comparable to the improvement achieved by a non-pharmacological life-style modification approach, such as exercise. While further work is needed based on our results, it seems that HD-RLS patients, at least the ones without advanced symptomatology, could employ an alternative and healthier control of their RLS symptoms if they adopt exercise.

According to the literature, a substantial placebo effect is a common outcome in most of the placebo-controlled RLS studies [25]. Interestingly, in our study, the patients who received placebo in the intervention period, exhibit improvements in the RLS severity only by 6%. It is noteworthy that this is the first trial that provide data regarding placebo-effect in uremic RLS patients. We hypothesize that the low placebo-effect that observed in our study may lie to the length of the treatment (6-months) as well as to the small sample size of the placebo group. We believe that in uremic patients, the maintenance of placebo-effect is limited when the intervention last for a long period of time such as the 6 months. Furthermore, a closer look on the individual’s placebo group data reveals that 3 patients experienced a slight worsening instead of relief in the RLS symptoms, fact that significantly reduce the potential placebo-effect of the current study.

The exercise-induced reduction in RLS symptoms could not be fully explained by the current data. Aerobic exercise training, apart from the recognized long term benefits it exerts on the cardiovascular system, exerts also acute benefits on the human brain by increasing the levels of an endogenous opioid called β-endorphin [26]. In a study of Esteves and colleagues in non-uremic patients, an inverse relationship was observed between β-endorphin release after exercise training and periodic limb movements index [27]. It is also known that the opioid system in the brain is involved in the pathophysiology of RLS [28] while treatments in the past using opioids successfully reduced the severity of the syndrome [29]. Interestingly, in a recent study, β- endorphins levels were found to be reduced in the thalamus of patients with idiopathic RLS [30]. That finding supports the possible mechanism for explaining the exercise benefit observed in our study, demanding further examination in the future.

Aerobic exercise training improved on patient’s physical performance, confirming previous data in HD patients with [10] or without [31] RLS, however the observed changes were statistical significant only on the within group comparisons and not compared to placebo group. The fact that exercise training improved parameters of the SF36 QoL questionnaire, LBM and muscle mass could explain the improved physical performance. Improving physical performance in a frail population, such as the HD patients, bears high clinical significance as the inactivity and the sedentary lifestyle are associated with poor QoL, comorbidities and increased mortality [31].

Previous studies have shown that dopaminergic agents can improve physical performance by reducing general fatigue in patients with Parkinson’s disease [8]. In our study, ropinirole administration did not show a measurable ergogenic effect on patients’ performance. This could be explained partially by the low ropinirole dosage used, in addition that, this study did not employ an “all out” exercise performance test that could reveal an underlying ergogenic effect (as stressing such patients to their limits is not advisable).

It is well known that the HD patients are characterized by a progressive decline in total lean body and muscle mass [32] due to uremia *per se* as well as the catabolic effect of the dialysis treatment [33]. Interestingly, recent data reveal that the HD patients with RLS may exhibit increased muscle atrophy compared to their free-RLS counterparts [5]. Aerobic [34] or resistance exercise training [35] could reverse or halt muscle wasting in HD patients, improving QoL and survival. Even though that LBM and muscle mass appeared to be improved in the exercise group after the intervention period (within groups comparison); no significant changes were detected compared to the placebo or ropinirole group. The low sample size seems to be the most logical explanation for the above findings.

According to the literature, DA could improve muscle mass through reduction in muscle fatigue [8], and by increasing growth hormone release and enhancing prolactin inhibition [36] and therefore promoting muscle anabolism. Both the exercise training and DA treatment could positively influence the level of LBM, however, the observed changes did not reach statistical significance levels compared to placebo arm. Improving LBM is something that should receive special attention from health care providers and the nephrologists since malnutrition and wasting are seen very often in the dialysis unit and are known to predict survival in this patient population [6].

Intramuscular lipid content is independently associated with diabetes mellitus in HD patients [37]. A sedentary life style lead to low physical fitness, which in turn has been linked to insulin resistance engrossing patients into a vicious circle of inactivity and muscle loss [38,39]. In our study, the CT analysis of the exercise group revealed evidence of improved muscle atrophy with concomitant reduction in the intramuscular fat deposits (extramyocellular lipids-EMCL) after the 6 months intervention (within group differences) (Table 5). However, the small sample size did not allow us to make safe conclusions regarding the superiority of exercise training compare to placebo in terms of reducing EMCL, as no significant differences were found between groups. The effect of exercise training in the reduction of fat infiltration in the skeletal muscles could be considered as an additional benefit towards the improvement of insulin resistance, and possibly lowering the risk for the early development of diabetes mellitus. The latter bears high clinical significance as the HD patients consist of a population with low-physical fitness levels [31] and with increased risk of developing insulin resistance and diabetes mellitus [40], especially those who have disturbed sleep [41].

Low QoL is associated with high mortality and high hospitalization rate as well as low adherence to medication in patients with chronic diseases [42]. Treatment with either ropinirole [43] or exercise training [10] in uremic RLS patients has been reported to improve QoL. According to the within group differences, both the DA and exercise groups earned a significant increase in the PCS of the SF36, while only DA group resulted to significant improvements in MCS of the SF36.

It is evident now that uremic RLS impairs significantly sleep quality and quantity [44]. In the current study, DA treatment significantly improved subjective sleep quality, confirming previous data in uremic RLS patients [4]. Both the DA and exercise training treatments did not improve daily sleepiness levels, confirming previous data [2,10] however, the majority of the patients had scored below the cutoff point (>10.0) that declare severe daily sleepiness.

The HD patients often suffer from depression symptoms [45] while higher scores in depression are observed in those with uremic RLS [46]. On the other hand, exercise training appears to have a rejuvenating effect decreasing depression levels in HD patients [31]. In our study, both the DA and the exercise training group reported significant improvements in depression score, whereas in contrast, depression levels appeared significantly worse in the placebo group after the 6-month period, clearly showing the negative effect that RLS can have if left untreated.

Even though the uniqueness of our study as well as the laborious methodology and highly skilled personnel required, a number of limitations remain. Due to the nature of the exercise intervention, a complete blinding in the exercise group was not possible. This could have induced a source of bias towards exercise intervention. In the current study we were not able to perform an overnight polysomnographic study in order to objectively evaluate sleep quality and quantity as well as to assess the periodic legs movements in sleep. In addition, no beta endorphins levels were assessed after training in order to further support our hypothesis. Even though the sample of our study was homogenous and recruited from the same dialysis unit, there were some numerical (not statistical) differences in some of the baseline characteristics between the three groups. In our study, the dosage of the ropinirole treatment was the minimum accepted in order to avoid any augmentation phenomena, however by not performing a dosage titrating we eliminated the comparability of our data to the studies using higher doses. Finally, residual renal function evaluation, a factor that may contribute to symptoms onset, was not included in the present study.

In conclusion, this is the first study comparing exercise training with dopamine agonists’ treatment in the amelioration of RLS symptoms, in patients with uremic RLS. Given the low and safe dosage of DA, we verified that both approaches were able to reduce RLS severity score with no side effects in either treatment group, in uremic RLS patients. It is noteworthy, that unsuccessful treatment of RLS symptoms with placebo led to worsening depression symptoms. Exercise training and low dosage of dopamine agonists are equally effective treatments in the amelioration of RLS symptoms in HD patients with uremic RLS. While further work is needed based on our results, it seems that HD-RLS patients, at least the ones without advanced symptomatology, could employ an alternative and healthier control of their RLS symptoms if they adopt exercise.

## Conclusion

A 6-month exercise training regime was as effective as a 6-month low dosage dopamine agonist treatment in reducing restless legs syndrome symptoms and improving depression score in uremic patients. Further research is needed in order to show whether a combination treatment could be more beneficial for the amelioration of RLS.

## Abbreviations

RLS: Restless legs syndrome; IRLSSG: International RLS study group; HD: Hemodialysis; DEXA: Dual energy X ray absorptiometry; DA: Dopamine agonists; QoL: Quality of life; MCS: Mental component summary; PCS: Physical component summary; UHL: University Hospital of Larisa; NSRI: North Staffordshire Royal Infirmary; STS: Sit-to-stand; CT: Computed tomography; LBM: Lean body mass; EMCL: Extramyocellular lipids.

## Competing interests

The authors have declared that no competing interests exist.

## Authors’ contributions

CDG: Analysis & Interpretation of the data, Drafting and Revising the article, Final Approval GKS: Conception and Design, Interpretation, Drafting and Revising the article, Final Approval CK: Design, Analysis, Interpretation, Providing intellectual content of critical importance to the work described GMH: Conception and Design, Drafting and Revising the article, Providing intellectual content of critical importance to the work described. EL: Analysis, Interpretation, Drafting the article. TK: Interpretation, Revising the article, Providing intellectual content of critical importance to the work described. YK: Design, Revising the article, Providing intellectual content of critical importance to the work described. IS: Conception and Design, Drafting and Revising the article, Providing intellectual content of critical importance to the work described, Final Approval. All authors read and approved the final manuscript.

## Pre-publication history

The pre-publication history for this paper can be accessed here:

http://www.biomedcentral.com/1471-2369/14/194/prepub
